# Regulatory Role of microRNAs Targeting the Transcription Co-Factor ZNF521 in Normal Tissues and Cancers

**DOI:** 10.3390/ijms22168461

**Published:** 2021-08-06

**Authors:** Emanuela Chiarella, Annamaria Aloisio, Stefania Scicchitano, Heather Mandy Bond, Maria Mesuraca

**Affiliations:** Department of Experimental and Clinical Medicine, University Magna Græcia, 88100 Catanzaro, Italy; aloisio@unicz.it (A.A.); scicchitano@unicz.it (S.S.)

**Keywords:** miRNA, ZNF521, transcription factors (TF), bioinformatics tools, cancer

## Abstract

Powerful bioinformatics tools have provided a wealth of novel miRNA–transcription factor networks crucial in controlling gene regulation. In this review, we focus on the biological functions of miRNAs targeting ZNF521, explaining the molecular mechanisms by which the dysregulation of this axis contributes to malignancy. ZNF521 is a stem cell-associated co-transcription factor implicated in the regulation of hematopoietic, neural, and mesenchymal stem cells. The aberrant expression of ZNF521 transcripts, frequently associated with miRNA deregulation, has been detected in several tumors including pancreatic, hepatocellular, gastric, bladder transitional cell carcinomas as well as in breast and ovarian cancers. miRNA expression profiling tools are currently identifying a multitude of miRNAs, involved together with oncogenes and TFs in the regulation of oncogenesis, including ZNF521, which may be candidates for diagnostic and prognostic biomarkers of cancer.

## 1. The Complex Regulatory Network between miRNAs and Transcription Factors

Most of the miRNA targets are transcription factors (TFs), which are critical in trans-regulating gene networks in many cellular systems [1]. TFs are proteins involved in regulating gene expression by binding to specific DNA sequences within the promoter regions to modulate such that they pre-transcriptionally activate or repress the expression of downstream genes [2,3,4,5]. miRNAs function to negatively regulate gene expression, targeting the posttranscriptional level by blocking translation and promoting degradation [6]. miRNAs and TFs can act cooperatively, exerting their regulatory functions on common target genes; in addition, they can influence the expression of each other [3]. 

**Figure 1:**
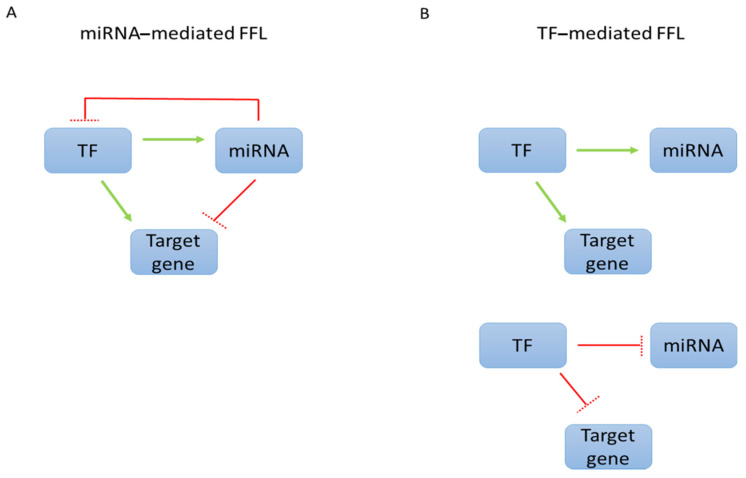
(**A**). Transcription Factors (TF) act directly on miRNA promoters and target genes for activation (green arrows) or repression (red blocked lines). (**B**). The combination of TFs and miRNAs can result in each regulating the other, giving repression of the TFs and target genes.

The miRNA–TF target regulation is orchestrated by a complex mechanism in which the expression of a miRNA can be regulated by TFs, and a gene encoding TFs can be inhibited by miRNAs [7] (Figure 1).

miRNAs and TFs act in a coordinated manner by forming two different autoregulatory feedback loops, the unilateral or reciprocal negative feedback loops and double-negative feedback loops, in which the expression of a component can be directly influenced by the presence or absence of the other [7]. In the first loop type, the expression of the TF is downregulated by the miRNA while the miRNA is activated by the TF. In the second type, the miRNA represses TF expression, directly, by binding to the 3′-UTR of target mRNA of the TF leading to its degradation or repression of translation, while the miRNA is itself transcriptionally repressed by the TF [7,8]. Additionally, TFs modulate gene expression by interacting with each other and binding to multiple sites for TFs in promoter and enhancer DNA, the so-called cis-regulatory modules (CRMs). The gene regulatory network of CRM-miRNA can be subjected to regulation by feed forward loops (FFLs). A FFL consists of input transcription factors and miRNAs, one of which regulates the other; both together are involved in target gene control. They can function according two models: in FFLs working in a miRNA-mediated manner, the miRNA may simultaneously repress the TF and its target genes; conversely, in TF-mediated FFLs, a TF is able to simultaneously activate or repress the miRNA and target genes—instead, in TF-mediated regulation is able to simultaneously activate or repress the miRNA and target genes (Figure 1). The activation of the two pathways produces opposite effects for maintaining the correct co-expression balance between miRNAs and target genes [7,9]. The complex dynamics of the regulatory network between TFs and miRNAs suggest that these molecules play a central role in many biological processes as well as in various diseases and cancers, when the regulatory axis is dysregulated [10].

In this review, we discuss miRNAs targeting the transcription factor ZNF521 to clarify the current literature, largely based on bioinformatic analysis. The interaction between different miRNAs and the transcription factor ZNF521 is at the center of complex regulatory networks during growth and development as well as during tumorigenesis. Currently, the investigation of the molecular mechanisms underlying the relationship between specific miRNAs and the ZNF521 target gene could aid in the understanding of tumorigenesis and provide molecular targets for alternative therapeutic approaches. 

## 2. The Transcription Factor ZNF521

**Figure 2:**
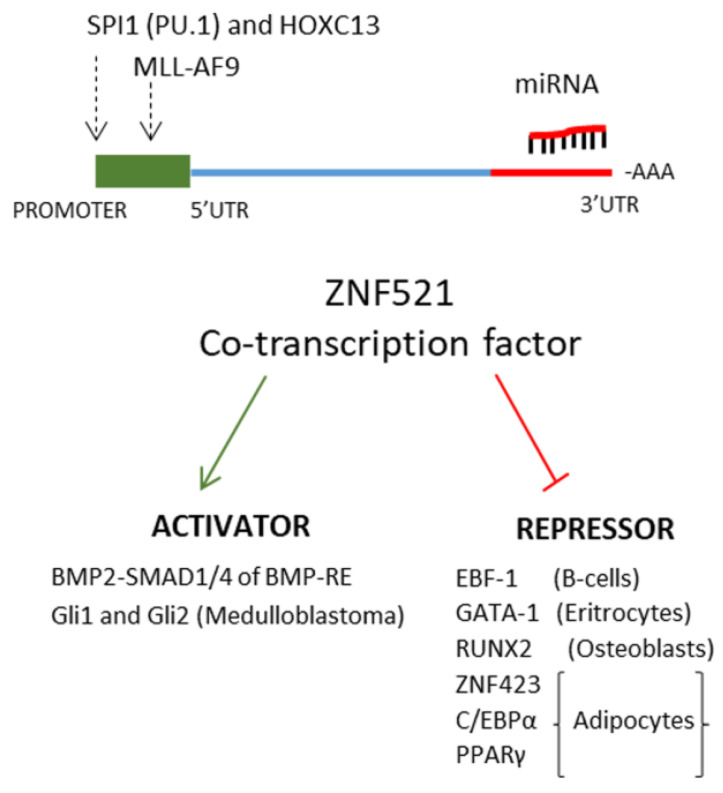
ZNF521 is transactivated at the promoter by the TFs SPI1-HOXC13 and MLL-AF9 or repressed by miRNAs binding the 3′UTR region. The ZNF521 protein can act as a co-activator with BMP2-SMAD1/4 (Bone morphogenetic protein 2- Small Mothers Against Decapentaplegic1/4) or Gli1/2 (Glioma-associated transcription factor1/2) alternatively as a repressor transcription co-factor with EBF-1 (Early B-Cell Factor 1); GATA1 (GATA-binding factor 1), RUNX2 (Runt-related transcription factor 2), ZNF423 (Zinc Finger Protein 423), C/EBP (CCAAT-enhancer-binding proteins) and PPARγ (Peroxisome Proliferation Activated Receptorγ).

Zinc finger protein 521 (human, ZNF521; mouse, zfp521) is a stem cell-associated transcription co-factor composed of 30 zinc fingers organized in 5 domains and an N-terminal transcriptional repressor motif that binds to the components of the nuclear remodeling and histone deacetylase (NuRD) complex [11,12]. hZNF521 shares high identity with the mouse zfp521 and considerable homology with hZNF423 [11,13]. ZNF521 protein was initially identified for its abundant expression in hematopoietic stem/progenitor cells and its levels are progressively reduced during myeloid differentiation [13]. ZNF521 can act as a repressor or activator of transcription depending on the cellular context. In the hematopoietic system, ZNF521 represses the erythroid differentiation program by inhibiting the activity of GATA-1 and attenuates the B-lymphoid differentiation of primary hematopoietic progenitors by antagonizing Early B-cell factor 1 (EBF1) [14,15,16]. hZNF521 plays a central role in maintaining the immature compartment of the mesenchymal system, where it has been shown to delay and repress both adipocyte differentiation, by inhibition of ZNF423, C/EBPα and PPARγ as well as osteoblastic differentiation and inhibiting RUNX2, in a complex interplay with EBF1 and ZNF423 [17,18,19,20]. Moreover, this stem cell-associated protein is involved in sustaining the phenotypic maintenance of articular chondrocytes in vitro [21]. In addition, hZNF521/mZfp521 are critical for neural development and bone formation (Figure 2) [22,23,24].

In addition to the role for ZNF521 in cell fate determination in different types of progenitor cells, this TF also plays a crucial role in cancer malignancies. The overexpression of this protein sustains clonogenic growth, migration and tumorigenicity of medulloblastoma cells, and recently it was demonstrated to interact with GLI1 and GLI2 proteins stimulating the trans-activation of the Sonic Hedgehog pathway in medulloblastoma [25,26]. Zfp521 has also been implicated in the induction of B-cell lymphomagenesis [27] and is significantly overexpressed in many acute myeloid leukemias (AMLs), particularly in those transformed by the oncogenic fusion protein MLL-AF9, coherently in MLL-rearranged AML cell lines and primary ex vivo MLL-AF9 expressing cells, ZNF521 depletion blocks AML progression [28,29,30,31,32]. A feed forward loop of transcriptional activation has been proposed where MLL/AF9 transactivates ZNF521 in AML leukemia [32].

In light of the remarkable multifunctional regulatory capabilities of ZNF521, in recent years, many studies have focused on identifying potential miRNAs able to target and modulate ZNF521 using novel bioinformatics platforms. The growing interest in miRNAs is linked to their ability to control the development and progression of tumors by targeting gene regulation at both the transcriptional and post-transcriptional levels, as well as transcription factors (TFs), such as ZNF521, which are considered important regulatory actors in solid and hematological malignancies. In addition to the role for ZNF521 in cell fate determination in different types of progenitor cells, this TF also plays a crucial role in cancer malignancies.

### 2.1. Interplay between miRNAs and ZNF521 in Pancreatic and Liver Cancer 

Pancreatic cancers were studied by bioinformatic in silico analysis [33] to identify novel mRNAs, lncRNAs, miRNAs, sdRNAs and piRNA. Next-generation sequencing was used to compare the transcriptome in normal pancreas and pancreatic cancer (PDAC) tissues. Evidence from bioinformatic approaches and RNA study technologies led to the identification of three novel miRNAs: miR-802, miR-2114 and miR-561, differentially expressed between PDAC and healthy control tissues, being down regulated in PDAC. Using Massive Analysis of cDNA Ends (MACE) and the omiRas web server, it was predicted that 16 genes were significantly upregulated in PDAC when there was the loss by post-transcriptional silencing of miR-802, being inversely correlated with target genes. ZNF521 was among PDAC upregulated genes together with other genes strongly related and influenced by ZNF521 in the mesenchymal stem cell commitment, such as PPARγ, RUNX2 and ZEB1 [17,18,34]. In silico analysis revealed that dysregulated transcription factors, including ZNF521, harbor binding sites in their 3′ UTRs for miR-802 with significant sequence complementarity [33]. Mesenchymal stem cells purified from adipose tissue (ADSCs) and bone marrow (BM-MSCs) are known for their potentiality to differentiate in multi-lineages toward mature osteoblasts, adipocytes, chondroblasts, and neuronal-like cells, where enforced ZNF521 has been found to have a transcriptional repressive role retarding adipocyte and osteoblast differentiation [17,18,19].

Compared to pancreatic cancers, hepatocellular carcinomas (HCC) express high miR-802, which is up-regulated compared to adjacent, non-tumor tissues. In 2020, Yang et al. [35] demonstrated that ZNF521 expression was abnormally down-regulated by miR-802 both in HCC tissues and liver cancer cell lines resulting in a malignant progression of the tumor. It was demonstrated by transcription reporter assays that miR-802 was physically involved in regulating ZNF521 expression binding to the miR-802 site in the 3′UTR of ZNF521 in HCCs. miR-802 overexpression significantly reduced the transcription factor expression in HCC cells with wild-type ZNF521 3′UTR compared to non-tumor tissues; on the other hand, anti-miR-802 induced an increase in ZNF521 mRNA and protein in HCC. In vitro gain- and loss-of-function assays of miR-802 in Hep3B and Huh7 cell lines in which endogenous ZNF521 levels were, respectively, lowest and highest, demonstrated that miR-802 was inversely correlated to the ZNF521 expression. miR-802 overexpression sustained cell proliferation, colony formation capability and cell viability in Huh7 cells, whereas miR-802 silencing exerts an opposite effect in Hep3B cells.

**Figure 3:**
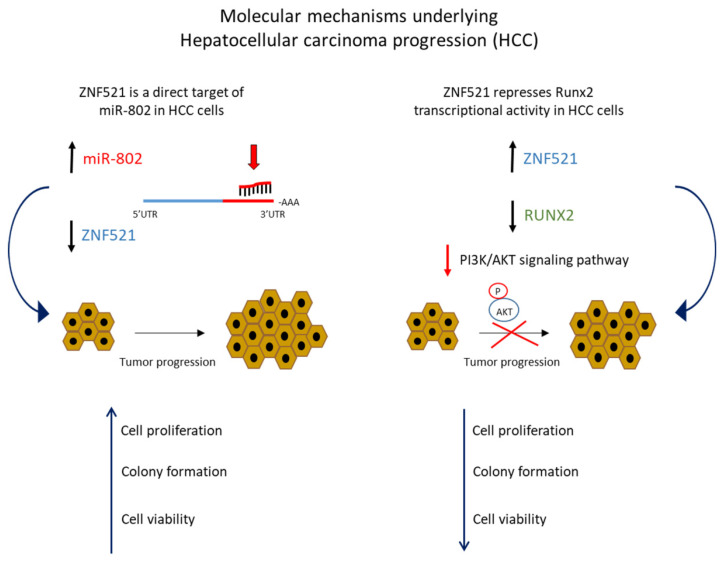
Molecular mechanisms by which ZNF521 promotes carcinoma progression in HCC (hepatocellular carcinomas). PI3K/AKT (Phosphatidylinositol-3-Kinase and Protein Kinase B); P-AKT (Phospho-Protein kinase B (PKB)). Black arrow indicates genes or miRNAs up or down regulated in HCC; blue arrow indicates the effects on cellular proliferation, colony formation and cellular viability in HCC sustained by miR-802 dysregulation or ZNF521 and RUNX2 gene expression impairment; red arrow indicates the pathway involved in HCC; blue curve arrow indicates the cellular effects produced by alterated genes or miRNAs expression; the arrow with red cross indicates the block in cellular proliferation by P-AKT.

In addition, it was shown in HCC cells that ZNF521, acting as a co-transcriptional repressor, binding Runx2 [36] and inhibiting its transcriptional activity involving PI3K/AKT signaling, blocks the tumor progression [35]. Runx2 is a bone-specific transcriptional regulator required during embryogenesis for skeletal development and is the main marker of osteoblastic differentiation [37,38]; the mouse protein, Zfp521, is able to bind Runx2 and antagonize its transcriptional activity, thereby controlling osteoblast differentiation from mesenchymal stem cells in vitro [39]. Moreover, Runx2 is expressed in many cancer cells promoting tumor progression [40]. In HCC cells, ZNF521 inhibits Runx2-related functions which has an important regulatory role in the promotion of cell migration and invasion by regulating MMP9 expression in HCC cells [41]. Furthermore, the inhibitory effect of ZNF521 in HCC cells is abolished by the AKT phosphorylation pathway, which has been shown to be involved in the mechanisms regulating the early stages of metastatic cancer progression [42]. Down-regulated ZNF521 expression may be relevant as a prognostic factor in malignant HCC, especially in the tumor TNM stage that is associated with poor prognosis. Understanding of the molecular relationship between miR-802 and ZNF521 could represent an advanced approach for in vitro diagnostics as well as a potential targeting strategy for treatment of HCC [35,43] (Figure 3).

In 2014, Bai and colleagues performed an integrated analysis of miRNA and mRNA in HCC cells; they compared miRNA and mRNA expression profiling in HepG2 cells and normal liver HL-7702 cells using next-generation sequencing technology. Bioinformatics analysis with specific enrichment tools (GO, DAVID and KEGG) allowed the identification of potentially dysregulated miRNAs correlated to target genes and their classification according to the biological processes and pathways in which they could be upregulated. In a complex network of mRNAs and miRNAs, ZNF521 seems to have a close relationship with miR-105 and miR-361-5p, although the molecular meaning was not addressed. ZNF521 was considered as a predicted target gene of these dysregulated miRNAs [44]. 

However, it must be considered that a single miRNA can target and regulate many mRNAs and an individual mRNA may be regulated by multiple miRNAs, resulting in complex regulatory networks [45]. Furthermore, miRNAs exert their role as if they were acting in an electronic circuit in which they function as switches that turn the circuits on/off [46], which govern proliferation, differentiation, the cell cycle, survival and apoptosis [47].

The cellular gene expression profile can be influenced by epigenetic events such as DNA methylation, histone modification, and miRNA-associated silencing during carcinogenesis. Human miRNA microarray analysis performed in eight HCC cell lines demonstrated that in Chromosome 19 the miRNA cluster (C19MC), which harbors 43 miRNA genes, was upregulated after combined treatment with DNA methylation and histone deacetylase inhibitors [48]. Unlike the 41 miRNAs in C19MC, 2 miRNAs (miR-517a and miR-517c) were able to induce G2/M blockage without causing cellular apoptosis and act as tumor suppressors in HCC. Using TargetScan and microcosm, five target genes were predicted for both miR-517a and miR-517c including ZNF521, protein tyrosine kinase 2 beta (PTK2B or Pyk2), ISL LIM homeobox 1 (ISL1), leucine-rich repeat transmembrane neuronal 3 (LRRTM3) and nuclear factor I/B (NFIB). Bioinformatics analysis revealed also that the 3′-UTR region of these predicted target genes contains a sequence to which miR-517a and miR-517c are able to bind. Based on these observations, ZNF521 can be considered a downstream target gene of both miR-517a and miR-517c, with a role in HCCs yet to be clarified [49].

The correlation between ZNF521 and miR-517a was also detected in studies focusing on low birth weight infants. miR-517a expression was highly expressed in the placentas of low birth weight newborns compared to normal birth weight newborns and its overexpression was implicated in the molecular mechanisms underlying the inhibition of trophoblast invasion. The 3′UTR sequence alignment and miRNA binding site prediction by TargetScan identified ZNF521 as a potential regulated gene targeted by miR-517a as well as other genes including SEMA3A and EIF4G3. Further studies are required to understand whether ZNF521 can be considered a downstream mRNA targeted by miR-517a to regulate trophoblast invasion [50].

### 2.2. Regulatory Role of miRNAs in ZNF521 Gene Expression in Gastric Cancer

ZNF521 also plays a central role in gastric tumorigenesis and progression through regulation by miR-204-5p [51]. MiR-204-5p exerts antitumor effects on tumor cells including breast, hepatocellular and gastric cancers [52,53,54]. Immunohistochemistry analysis demonstrated that the ZNF521 protein was highly expressed in gastric cancer tissues (GC) compared to adjacent normal tissues. In addition, ZNF521 expression correlates with larger gastric tumor size, advanced TNM stage and local lymph node metastasis. Kaplan–Meier plots revealed that high expression of ZNF521 was correlated with a shorter overall survival time. ZNF521 ectopic expression enhanced the proliferation, migration, and invasion of gastric cancer cell lines. Rescue experiments in vitro revealed that silencing of ZNF521 antagonized the malignant phenotype. The phenotypic and molecular changes modulated by gain- or loss-of ZNF521 gene expression are strongly related to the tight control mediated by miR-204-5p. The binding to the 3′UTR of ZNF521 of miR-204-5p predicted by TargetScan was verified by transactivation luciferase assays [51]. The expression levels of miR-204-5p strongly (R=0.65) negatively correlated with that of ZNF521 in clinical samples. In addition, the MKN gastric cell line, representing the ZNF521 low expression model, exhibited a significant ZNF521 upregulation when 28-miR-204-5p inhibitors were introduced compared to overexpressing ZNF521 AGS cells and those transfected with miR-204-5p mimics. In gastric cancer cells, low expression of miRNA-204-5p facilitates the proliferation, migration, and invasion of GC cells permitting an upregulating ZNF521. Understanding the molecular relationship between ZNF521 expression in GC cells could open new horizons for the identification of clinical-pathological features and molecular prognostic markers for gastric cancer patients [51].

### 2.3. Role of miRNAs in Regulating ZNF521 Gene Expression in Female Cancer

From an integrative model of miRNA and mRNA expression, it was found that ZNF521 was one of the genes which plays a protective role in the patients affected by breast invasive carcinoma (BRCA) who had undergone radiation therapy [55].

The results showed that nine mRNAs (CYP20A1, MAVS, ASXL1, KLF3, ZNF70, H6PD, ZMYM2, ICA1 and ZNF521) exerted a protective effect while five miRNAs (hsa-mir-181a-2, hsa-mir-582, hsa-mir-125, hsa-mir-874, has-mir-222) and eight mRNAs (CPA4, AIMP1, PAX6, CCDC34, IER5, PERP, RRM2B, VDAC1) were considered as high-risk genes [55].

In ovarian endometriosis ZNF521 appears also in a complex integrated miRNA–TF–gene regulatory network [56], it is possible that a deregulation in the relationship between ZNF521 and ovarian tissue-specific miRNAs could induce molecular changes causing endometriosis. Specifically, an integrative analysis of miRNA and mRNA indicated ZNF521 as a target of the transcription factors FOXC1, FOXL1 and GATA1 which act on the miR34-family, miR133b and miR-202-3p [57].

### 2.4. miRNA and Gene Regulation in Urologic Malignancies 

ZNF521 expression is influenced by miRNAs also in urological malignancies, including bladder cancer (BC). A microarray platform was used to investigate the profiles of miRNA expression in BC compared to normal bladder urothelium. ZNF521, together with TMCC1, TNIP1, VSTM2B, ISL1, emerged as target genes of miR-517a, the expression of which is significantly enhanced in bladder cancer [58,59]. miR-517a functions as a tumor suppressor in BC cells; the treatment with a demethylating agent, 5-aza-2′-deoxycytidine (5-Aza-dc), sustained miR-517a restoration, inhibiting cell proliferation and inducing apoptosis in BC cell lines [60]. In addition, in hypoxia conditions altered miRNA expression pattern in non-muscle-invasive bladder cell lines (RT4 and RT112) induced a significant upregulation of several miRNAs including miR-517a [61]. However, further studies are needed to dissect and analyze the biological processes between the miR-517a gene expression pattern and ZNF521 in order to understand the implications in bladder cancer oncogenesis and possibly provide the basis for discriminating between non-invasive muscle bladder cancer low grade (NMIBC) from high grade and muscle invasive bladder cancers.

### 2.5. Association between miRNAs and ZNF521 Idiopathic Pulmonary Fibrosis

Idiopathic pulmonary fibrosis (IPF) is a severe scarring chronic lung disease characterized by a progressive and irreversible impairment in lung function [62]. The molecular signature of pulmonary fibrosis displays differentially expressed genes (DEGs) and differentially expressed miRNAs (DE-miRNAs), which control gene expression. Web-available microarray data [63] from IPF and non-diseased control lung tissue samples were used to identify miRNAs and genes associated with this form of idiopathic interstitial pneumonias. 

The microRNA microarray dataset GSE32538 and the mRNA datasets GSE32537, GSE53845, and GSE10667 identified a role for ZNF521 in the biological processes of IPF. ZNF521 is differentially expressed in IPF and can be deregulated by specific miRNAs (hsa –mir-19b-3p, hsa -mir-30a-5p, hsa -mir-30d-3p, hsa -mir-92a-3p), which have been recently identified as regulators for genes in pulmonary fibrosis [64,65].

In addition, protein–gene interactions analyzed by the STRING web tool revealed the relationship among 67 common differentially expressed genes in IPF. This analysis identified nine hub nodes (COL1A1, MMP1, COL3A1, TNC, SPP1, MMP7, POSTN, ITGB8, and COL6A3) and the proteins with the highest degree of interaction connected to them. In this network ZNF521, together with LEPREL1, TGFB3, COL15A1, COL17A1, and TNC, interacts with the hub node COL1A1 [64,65].

In lung epithelial cells and fibroblasts, these miRNAs are predicted to exert a profibrotic role targeting COL1 gene expression; for this reason, COL1 was reported as the first of the top nine nodes in the protein–protein interaction network of DEGs. The biological validation of miRNA-mediated regulatory networks among the DE-miRNAs and DEGs could be interesting to identify new potential biomarkers for the prediction of IPF progression [63].

### 2.6. miRNAs Targeting Zfp521 in the Neural System 

The mouse protein Zfp521 is highly expressed in neural stem cells playing a role in the ontogenesis and in the regulation of specialized functions in the central nervous system [66]. Zfp521 has been implicated in the transition of embryonic stem cell differentiation into neural progenitors [22]. MiR-9 targets Zfp521 to promote the differentiation of bone marrow mesenchymal stem cells (MSCs) into neurons. MiR-9 expression was detected in neurogenic areas of the brain and is involved in the regulation of neural differentiation from embryonic stem (ES) cells as well as from neural stem cells [56]. When BMSCs were induced to differentiate into neuron-like cells the expression levels of Zfp521 declined; in this context it was observed that miR-9 was capable of restoring and promoting neural differentiation via targeting Zfp521, consistent with the 3′-UTR of Zfp521 containing one potential miR-9 binding site (nt 1188–1193) [23].

The human protein ZNF521 was found to be a target gene for miR-381 in the striatum of macaques during acute-SIV infection and chronic administration. Within the neuroimmune-modulatory microRNA profile miR-381, whose expression was significantly increased, belongs to a cluster of five miRNAs (miR-485, -382, -134, -381 and -539) located close together on chromosome 7 in Rhesus and chromosome 14 in humans, in a region particularly enriched for the H3K227Ac histone mark and, therefore, with high transcriptional activity associated with Fos, Jun, FoxA1, Foxa2, Sp1 and GATA [67]. Further analysis is needed to understand the relationship between ZNF521 and miR-381 in this context.

**Figure 4:**
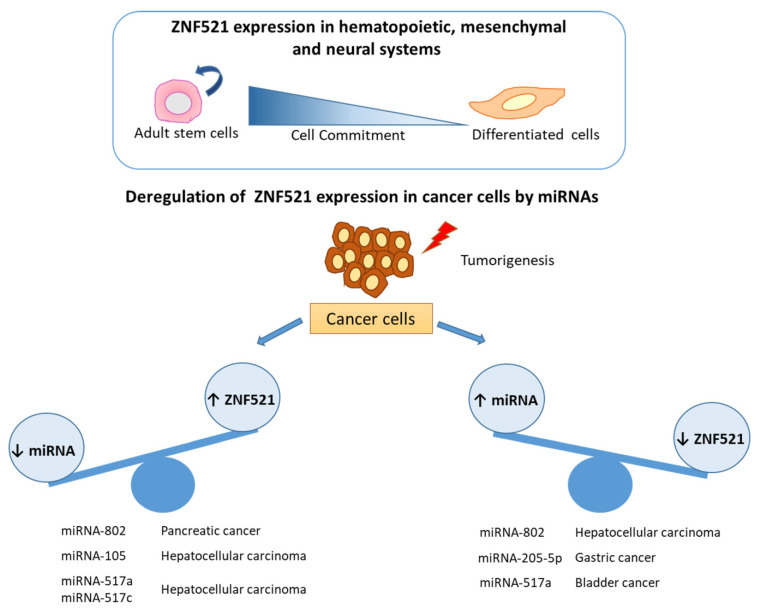
MiRNA targeting the 3′UTR of ZNF521 in different cancers. Thunder symbol indicates cancer cells, light blue lines indicate the unbalance between specific miRNAs and ZNF521 expression in different tumors.

The miRNAs targeting ZNF521 in these different cancers and cellular systems have been reassumed in Figure 4.

## 3. Conclusions 

In this review we discuss the current knowledge concerning miRNAs targeting the transcription factor ZNF521, as yet not described in this context. We focused the attention on data from computational miRNA prediction tools identifying putative miRNAs targeting ZNF521 and cellular/molecular experimental models validating the bioinformatics data and clarifying the mechanisms by which miRNAs can also regulate ZNF521 expression in gene regulatory networks. ZNF521 is a stem cell-associated transcription co-factor with recognized regulatory functions in the immature compartment of the hematopoietic, mesenchymal and neural systems. 

However, it is still to be investigated whether ZNF521 targeting miRNAs plays a role in inducing the expansion of hematopoietic stem cells where this transcriptional factor is abundantly expressed [68]; this could be important for ex vivo expansion of hematopoietic stem cells for clinical applications.

Taken together, ZNF521 can be considered as a cancer-related gene which is aberrantly expressed in MLL-rearranged AML and involved in the induction of B-cell lymphomagenesis. ZNF521 expression is also deregulated in solid tumors including medulloblastoma cells with an increased tumorigenic potential as well as in other solid tumor types including hepatocellular carcinoma, gastric cancer cells, invasive bladder transitional cell carcinoma as well as breast and ovarian cancers. Integrated analysis of high-throughput systems revealed a close functional inverse relationship between the specific miRNAs and ZNF521 expression levels comparing cancers and controls. The dysregulation of the miRNA–ZNF521 axis may be crucial for the acquisition of cancer-initiating properties by stem cells and for the molecular changes occurring during cancer progression. Identifying new miRNAs targeting ZNF521 and understanding the molecular basis of their interaction will support the search for novel biomarkers or development of advanced therapeutic strategies.
